# Remarks on the Diseases of Tropical Climates; with Some Calculations Relative to Statistical Medicine

**Published:** 1823-04

**Authors:** 


ART. IV.—*Remarks on the Diseases of Tropical Climates; with some Calculations relative to Statistical Medicine.* By an ARMY MEDICAL OFFICER.

TILL of very late years, the young practitioner, proceeding to a tropical country, was placed in a very embarrassing situation. Of books, professing to afford him instruction, there was no deficiency; but the quantity of exact and accurate information respecting the diseases he was likely to meet and contend with, was generally very inadequate to the importance of the occasion. The progress from ignorance to experience, I could delineate; and it would be a painful, but not an useless task.

The zeal, however, for improvement which animates every rank of our profession at home, has inspired also our brethren abroad; and we have lately seen added to the list several most valuable works, the result of an enlightened and extensive experience. When I mention the names of Jackson, Macgrigor, Bancroft, Irving, Burnett, Bampfield, Johnson, Ballingal, Marshall, and Chisholm, the reader will readily agree with me, that tropical medicine has called forth its due share of talent.

Notwithstanding all that has been effected for the improvement of tropical medicine, much still remains to be done; and, when we reflect on the work of desolation which is so frequently carried on in our colonies in the East and West Indies, by the destructive jungle and yellow fevers, by cholera, hepatitis, and dysentery,—when we consider that, by no exaggerated calculation, seven out of every eight of the British army who perish by disease, are cut off in our colonies abroad, chiefly within the tropics,—we must be impressed with a high idea of the importance of the subject of these remarks. I could enlarge on this topic, but deem it for the present unnecessary. The light which pathology must derive from a careful examination of the bodies of the victims of disease in these countries, I consider an object deserving the highest consideration. I am far from meaning to infer that this has been neglected, or wishing to throw the smallest blame on our tropical brethren: we have the fullest proofs, in their works, that neither their zeal nor their exertions have been deficient. We have much oftener occasion to complain of the partiality of the views of medical men, than of a want of zeal for investigation. Many remarks and observations, which at the moment appear trifling and insignificant, may, in the progress of science, assume an aspect of the highest interest. For instance, in fevers, morbid alterations of the brain, the liver, and the stomach, have been again and again assigned as their proximate causes. The former two, as well as the lungs, have been found occasionally disorganized; the latter more generally so; yet the difference of opinions still existing among medical men clearly proves that the question remains undetermined. Other doctrines are daily advanced, and we find that, in our former investigations, some circumstances have been overlooked, which, had they received their due share of consideration, would, in a great measure, have enabled us to determine the validity of the new opinions.

Of all the sects which in the present day exist in medicine, the Broussaists are undoubtedly the most zealous and the most clamorous. That the group of symptoms we designate by the word Fever owes its origin to a primary inflammation of the mucous membrane of the stomach and intestines, is an opinion claimed by M. Broussais as exclusively his own,—erroneously, I believe; for similar sentiments are to be found in preceding authors. The name and doctrines of Broussais are only partially known in this island, but they have excited among our Gallic neighbours a revolution in medical opinion, even more complete, and far more salutary, than that effected by Brown in Italy and Germany. On the justice of these opinions we are not at present called upon to decide: I am anxious, however, to direct the especial attention of my brethren to a careful examination of the internal surface of the intestinal canal. This subject has been too much overlooked in post-mortem examinations. I have frequently been surprised to find extensive ulceration of the small intestines, where none had been suspected during life; and where neither the feelings of the patient, nor the symptoms of the disease, indicated any such affection. I have several times had occasion to observe cases of fever, with all the symptoms of typhus, in which ulceration had taken place through almost the whole extent of the ilium. It is useful to observe, that the peritoneal coverings frequently evince little or no appearance of change, while very extensive disease may have taken place in the mucous membrane. It is necessary to pay minute attention to the internal surface,—to observe the nature of the adhering matter,—and to compare carefully the diseased with the healthy parts.

The smaller West-Indian islands may be divided into high and low, with respect to surface; and into argillaceous with granitic basis, and coralline, with all its varieties, respecting structure. The larger ones possess every variety of surface and structure. Some are more remarkable for marsh than others, and those unhappily so distinguished are the most inimical to the human constitution. In the higher islands, the great agent of insalubrity is the irregularity of their temperature; the humidity of their atmosphere proves also another source of unhealthiness. The windward islands are more marshy, and consequently more unhealthy, than the leeward. The year is divided into two portions, the dry and wet seasons. The dry season is the portion of the year between the beginning of December and the end of April. In its general course, it is pleasant and healthy. The rainy season comprehends the two last months of summer, all autumn, and generally the first month of winter. The quantity of rain that falls is extremely variable. The mean temperature of the islands, at the sea-side, in the shade, according to Dr. Chisholm,* is 84° Fahr.; at the accessible parts of the higher mountains, about 60°. It is to be suspected that, in the present instance, Dr. C. has fallen into some error of calculation. Humboldt and Dr. Brewster have assumed 81.5 as the mean temperature of the equator. At Trincomalee, situated in $8\frac{1}{2}$ north latitude, the mean temperature for two years was $80\frac{5}{12}$: I therefore believe Dr. Chisholm's estimate to be too high. The thermometer, he says, almost uniformly exhibits the following movements: at seven A.M. the mercury begins to rise, and continues to do so till one P.M.; from which time till seven A.M. it is again stationary. The principal deviation from this routine is caused by some remarkable change in the atmosphere, such as rain with strong winds: the greatest change he has observed from this cause is 10°. The thermometer, exposed to the direct rays of the sun, has risen in ten minutes to 130°, or 42° above its stationary point in the shade; but 30° he considers the medium difference. Dr. Chisholm, it is presumed, must have used a thermometer with a metallic scale, because a small glass thermometer, with an ivory scale, never indicates a difference greater than a few degrees between the most direct exposure to the sun and the shade in a tropical country.

There is no subject, I believe, involved in such utter obscurity as the causes, direct or indirect, of most diseases; yet, were we to give credence to former writers on this subject within the tropics, nothing is so simple or so easily explained. When

---

* See a *Manual of the Climate and Diseases of Tropical Countries.* By COLIN CHISHOLM, M.D. F.R.S. &c.

their hypotheses, however, are soberly and minutely examined, we find them, like most other conjectures, liable to innumerable objections. It is not my intention, at the present moment, to enter more fully into this subject: I conceive that the time of your readers may be more profitably occupied by presenting them with the result of some inquiries into the prevalence and mortality of certain diseases in the East and West Indies, compared with Great Britain.

The documents which have been published respecting the absolute and relative mortality of particular countries and diseases, are, it must be lamented, in a great many instances, more or less defective. On this highly important and interesting subject, much may be expected from the increasing exertions of the eminent individual at the head of the army medical department. By establishing excellently well-constructed returns, and by encouraging the medical gentlemen of the army to register whatever important facts may occur to them, much useful information may be made known to the public, that would otherwise be buried in oblivion.* Medical topography will, by these means, soon become better known, and more generally studied. In the calculation of disease, I would recommend Mr. Marshall's tables, as the most perfect I am acquainted with. By tables of this kind, we are at one view furnished with important information; the construction is extremely simple; and the mean mortality per cent. to the whole strength, appears to me by far the most determinate manner of statement, and is the one I shall constantly make use of in the course of these remarks.

Dr. Hunter informs us, that the mean mortality of the whole troops in the West Indies for three years and a half, commencing from the year 1780, was about 25 per cent. It may be remarked, that these troops were not only unacclimated, and employed in active service, but were almost entirely composed of newly-raised regiments. In the same islands, the mortality among the British troops for seven years, (viz. from 1796 to 1802,) was 21.3 per cent per annum. The highest mortality was in 1796, when it amounted to 40.5; the lowest in 1802, when is was 11. For the same series of years, the mean annual mortality among the negro soldiers was 5.7 per cent; and it is remarkable that, in 1796, it was only 3, being the lowest rate of mortality during the above number of years.

Dr. Jackson has furnished us with a statement of the mortality

---

* We cannot avoid expressing our concurrence in these sentiments; and we take the opportunity, both of acknowledging our obligations to the Director General, and to those army-surgeons who have already favoured us with communications. We trust that our Journal may be considered by them as a fit medium for giving to the public many of those valuable observations, which, as our Correspondent observes, "would otherwise be buried in oblivion."—Editors.

in the leeward and windward islands and colonies, from 1803 to 1814. The mean annual proportion during these years is much smaller than in the former series, being 12.6 per cent. The highest mortality was in 1810, when it amounted to 20.5; the lowest 1814, when it was 5. The mean annual average from 1796 to 1814, will be nearly 16-9 per cent.

In some particular parts of the eastern hemisphere, the ratio of mortality is sometimes as great as the highest rate we have just stated; but the mean annual mortality for a number of years is considerably less. I shall present the reader with a few specimens of the mortality of different regiments; and I consider them as curious and important, in exhibiting the steady healthiness of some stations compared with the unhealthiness of others. Some of the most remarkable are selected from Mr. Marshall's work on the Medical Topography of Ceylon. The 19th regiment of foot landed on that island on the 28th April, 1796, and, with the exception of one year on the peninsula of India, remained there till December 1819, when it was embarked for England. During this period, the mean mortality per cent. per annum was 7.6; invalided, 2.7. In Kandy, in 1803, the mortality was 40 per cent. At Trincomalee ($8\frac{1}{2}^\circ$ N.L.) in 1804, they lost 19.9 per cent: the lowest mortality at that station was 5.7. At Colombo ($5^\circ$ N.L.) the highest mortality was 5 per cent., the lowest 1.7; or, at Colombo during nine years, the mean annual proportion was 2.8. At Trincomalee, for the same number of years, it was 8.5. In 1820, they were partly at Chatham and partly at sea, their loss was 4.7: in 1821, they were stationed at Winchester, and lost 1.1 per cent.

The 73d regiment, in the Kandean provinces, in 1818, lost 41.2 per cent.; next year, 28.2; and in 1820, partly at Galle, partly at Trincomalee, they lost 7.1; making a mean proportion of 25.5 per cent. per annum; invalided, 5.3. According to this ratio, a regiment would require to be renewed every 3.9 years.

In the same years, the 83d regiment were partly in the Kandean provinces, partly at Colombo; the mean annual mortality was 8.6. During the year, the mortality at Colombo was 2.5; that in Kandy, 6. During three years, the women of the regiment died at the rate of 5.3 per cent. annually; the children at 12.3.

The 45th regiment were in Ireland from 1815 to 1819, where the mean annual mortality per cent. was a little more than 1.2. They arrived in Ceylon in 1819: the mean annual mortality, during that and the subsequent year, was 4.7. The women of the regiment died in the proportion of 8 per cent.; the children, 53.4.

A remarkable contrast to the rate of European mortality in

India is afforded by the returns of the Malay regiment in Ceylon, from 25th August, 1811, to 31st December, 1820: the mean annual loss per cent. was 3.4; but in one year, at Trincomalee, they lost 11.4: deducting that year, would reduce the mean mortality to 2.5. In 1818, when the 73d regiment lost 41.2 per cent., the Malays on the same service lost only 4.4. In different stations, the mortality was occasionally as low as 0.9. Four years at Galle and Colombo, give a mean annual loss of 1.4 per cent.; three years in the Kandian provinces, give 4.5.

On a comparison of ten regiments, stationed in Java, Ceylon, Madras, and Bengal, for a considerable period, we find the mean annual mortality to be about 10.2 per cent.: but the rate of mortality is probably, as an average, too high; as, in one regiment included in the calculation, the mortality was 25.5,—in none of the others did it exceed 9: deducting that regiment, the mortality will be about 6.3. In England, I have occasion to know that, among the same class of men, it amounts to a little more than 1 per cent. In North Britain, for three years, from the 21st June, 1817, to 20th June, 1820, it was only $\frac{78}{100}$, or one death in 128.

Observers in general have assured us that, amongst any given number of men, a greater proportion of mortality occurs during the first years of residence in warm climates, than at any subsequent period. From Dr. Chisholm's remarks, this appears to be remarkably the case in the West Indies; and, as far as my information extends, I believe it to be true also with respect to the East, although not very remarkably exemplified in the regimental returns. In one regiment only do we find it particularly striking. The Royals, during their first year, as we are informed by their surgeon, Mr. Ballingal, lost by disease 21.3 per cent.; next year, their loss was only 4. This regiment was chiefly composed of boys and very young men, who, from Mr. B.'s statement, suffer more from climate than adults. Selecting a station considered as extremly salubrious, where no endemic causes of unhealthiness exist, and where the diseases in one year compared with another bear exactly the same character, we shall find a variation of mortality in the reports for which it is difficult to account. The 19th regiment, during their first year at Colombo, lost 4.4 per cent.; next year, 2.2. In 1807, at the same station, they were joined by 369 recruits, yet the deaths that year were only 2.5 per cent; in 1809, when they were joined by 70, the mortality was 5 per cent.; in 1812, they were joined by 105, and lost 1.7 per cent. The 34th regiment had been two years in India in 1805; from that year to 1810 they were constantly in the same station, and the mortality varied in the following ratio:—4.7, 11.3, 9.2, 3.2, 2.9.

Disease, in warm countries, appears generally under the form of dysentery, fever, and hepatitis; and, with a view of exciting attention to this interesting subject, I shall enter, somewhat fully, into a statement of the proportion which these affections bear to all other diseases.

On examining the returns of the 83d regiment for three years in Ceylon, I found that the mean annual mortality during that period had been 8.5 per cent.: the loss from fever, dysentery, and hepatitis, was 6.7; other diseases, 1.8 per cent. The 59th regiment arrived in India in April 1806: their loss, till the 31st December, 1806, was 5 per cent. At the same rate of mortality, this would have given 6.7 per annum. The following are the proportions:—Fever, 0.4; dysentery, 2.9; hepatitis, 1.1 = 4.4; other diseases, 0.6 = 5. An analysis of Mr. Ballingal's tables for six years presents us with the following results:—Mortality from fever, 0.6; dysentery, 5.5; hepatitis, 0.5 = 6.6; other diseases, 1.2 = 7.8. Pulmonic affections were little more than one in a thousand; rheumatism, one in 1325. At the risk of being thought tediously minute, I shall present the reader with an analyzed abstract of Mr. Marshall's tables of the mortality of the troops in the Kandean provinces for three years.

| Diseases. | Europeans. *Mean ann. Mortality per Cent.* | Malays. *Do.* | Caffres. *Do.* | Indians. *Do.* |
|---|---|---|---|---|
| Fever | 7.4½ | 1.6 | 0.1 | 0.5½ |
| Dysentery | 3.3 | 1.1 | 1.1½ | 1.9 |
| ———, with diseased Liver | 1.1½ | — | — | — |
| Hepatitis | 0.2½ | — | 0.4 | 0.2 |
| Total | 12.1½ | 2.7 | 1.6½ | 2.7 |
| Cholera | 0.8 | 0.8 | 2.0½ | 1.3 |
| Variola | — | — | 1.1½ | 1.2 |
| Pulmonic affections | 0.1½ | 0.4 | 1.1½ | 0.0½ |
| Other diseases | 0.7 | 0.4½ | 1.3 | 1.0½ |
| Total ann. Mortality per Cent. | 13.8½ | 4.3½ | 8.2 | 6.3½ |

During one of the years included in the above return, cholera was epidemic throughout all India; during another, variola prevailed in Ceylon among particular classes: but these two diseases, occurring only once during a long series of years, are not to be admitted into a general calculation.

The deaths among the Europeans is greater than the usual mean; but the proportion of fever, dysentery, and hepatitis, to other diseases, is probably in the usual rates. The Caffres, it appears, are almost entirely exempted from fever, while they suffer in a very great proportion from pulmonic complaints.

From other tables, it appears that only four Europeans died in the Kandean provinces with symptoms of phthisis pulmonalis during six years, being about one in every 1220 per annum. In one of these fatal cases, the pectoral symptoms were combined with dysentery; and in another, with abscess of the liver,—which latter may have been the primary affection, as well as cause of death.

There are few diseases so very rarely to be met with as pulmonary abscess without tubercle. Laennec has only seen one instance in which the collection was at all considerable. Pneumonic inflammation is very rare among Europeans in the East Indies; in the West, it appears to be more frequent. I have already alluded to the proportion in which phthisis occurred in Ceylon, (one in 1220:) in Great Britain, the mortality from consumption is estimated at one in 224 of the whole population. In Geneva, Dr. Chisholm states it at one in 521.*

I have, however, to remark, that, among the Caffres in Ceylon, consumption is extremely frequent. These men were chiefly brought from the eastern coast of Africa, and, for the most part, from the neighbourhood of Mozambique. They are very liable to the tubercular disease in all its forms; the mesenteric glands are frequently found enlarged, and filled with tuberculous matter; the peritoneal covering of the intestines thickly beset with tubercles, and the folds agglutinated together. Malays are also much more subject to pulmonic inflammation than Europeans.

The existence of a contagious fever has hardly ever been suspected in the East Indies, although fevers of a nature as malignant and as destructive as those of the West are occasionally produced. I could enumerate many instances where it attacked almost every one exposed to the influence of its cause, but shall only notice one. At Kottabawa, in Ceylon, from the 12th July to the 20th October, 1818, 252 Europeans out of 254 were attacked with fever: of this number, 193 died; and the constitutions of those who escaped were so much impaired as to render it probable that few, if any, of them would ever completely recover. Sixty Caffres were stationed at the same post, but not one had fever. The indigenous inhabitants also suffered much from fever, but its extent among them could not be ascertained. The causes which, in my opinion, prevent any idea of contagion being entertained in the East, are the following:—In particular situations, and even whole districts, fever is almost entirely unknown: in others, at certain seasons, it is

* Lib. cit.

constantly present, in a milder or more violent degree. If, as is frequently the case, a patient affected with fever be removed from the latter to the former, the disease is never in any instance communicated. Situations which have proved dreadfully unhealthy one year, are not necessarily so the next: it probably happens that for four or five years they remain comparatively healthy, intermittents or slight remittents only prevailing. Again, in its season, the disease breaks forth with violence,—continues for a time, and then disappears. It attacks strangers with much more violence than the inhabitants accustomed to the climate, and some classes appear almost entirely exempted.

An inquiry into the influence and action of mercury in the treatment of tropical diseases, together with a fair and candid statement of the comparative success of the different methods of practice adopted by different practitioners, would afford much interesting matter for discussion. Dr. Jackson seems to think that the diminution of mortality in the West Indies, during the latter years of the period included in his returns, was chiefly owing to the free employment of venesection.

*Edinburgh; November* 1822.

